# Systematic Review on Protocols of Coenzyme Q10 Supplementation in Non-Surgical Periodontitis Therapy

**DOI:** 10.3390/nu15071585

**Published:** 2023-03-24

**Authors:** Cordula Leonie Merle, Carina Lenzen, Gerhard Schmalz, Dirk Ziebolz

**Affiliations:** 1 Department of Prosthetic Dentistry, UKR University Hospital Regensburg, 93053 Regensburg, Germany; 2 Department of Cariology, Endodontology and Periodontology, University of Leipzig, 04103 Leipzig, Germany

**Keywords:** ubiquinone, antioxidants, nutritional supplement, periodontal disease, non-surgical treatment, periodontal treatment outcomes, adjunctive periodontal therapy

## Abstract

This systematic review focuses on the different study protocols on CoQ10 as an adjunct in non-surgical periodontitis therapy. The study protocol was developed following PRISMA guidelines and was registered in PROSPERO (CRD42021156887). A sensitive search up to January 2022 considered MEDLINE via PubMed and Web of Science, Embase, Web of Science Core Collection via Web of Science, Google Scholar, Cochrane CENTRAL, WHO (ICTRP), ClinicalTrials.gov, and grey literature. Randomized controlled (SRP with/without placebo) clinical trials (RCTs) on all types of CoQ10 administration were included. The primary outcome was probing pocket depth (PPD). Secondary outcomes were bleeding on probing, clinical attachment loss, and gingival and plaque indices. Twelve RCTs with local and five with systemic CoQ10 administration were included. The study protocols were heterogeneous. Local CoQ10 administration was performed once or several times in a period up to 15 days. Systemic CoQ10 was applied twice or three times daily for six weeks up to four months. The reporting quality was low, including missing information about CoQ10 doses. Risk of bias was high or unclear. About half of the studies reported significant group differences for PPD. Until now, no statement on the effectiveness of CoQ10 in non-surgical periodontitis therapy is possible. Further high-quality RCTs are necessary and should consider the protocol recommendations of this review.

## 1. Introduction

Periodontitis, a multifactorial chronic inflammatory disease, is based on disturbing interactions between dysbiotic biofilm and the inflammatory host response [1,2]. The patient’s immune system itself leads to tissue destruction due to an exacerbating immune-inflammatory host response [3,4].

The primary therapeutic strategy refers to the disruption of the biofilm by mechanical scaling and root planing (SRP). This mechanical non-surgical treatment has been established as the gold standard [5,6]. However, success depends on a variety of factors, especially the patient’s individual host immune response [2,7,8]. For this reason, the administration of pharmaceutical agents along with mechanical debridement is of interest [3,9]. Until now, established adjunctive therapeutic approaches target the microbial level. Antimicrobial agents such as antibiotics [10,11] or antiseptics (e.g., chlorhexidine) [12,13,14] have been applied to increase the effectiveness of biofilm reduction. The clinical relevance of these adjuvants remains questionable due to limited differences in outcome [11,13] and possible side effects as well as potential antibiotic resistances [10,12,15]. 

Accordingly, other approaches are increasingly generating scientific interest, e.g., probiotics [16,17]. Recently, coenzyme Q10 (CoQ10) as a supplement for non-surgical periodontitis therapy has been proposed [18]. CoQ10 is an endogenous component of the mitochondria and plays a key role in energy production in the respiratory chain through oxidative phosphorylation. As an antioxidant, it affects the fluidity and stability of the cell membrane and lipoproteins [19,20], protecting them from oxidative damage by free radicals [21]. Additionally, CoQ10 has anti-inflammatory properties, as it suppresses the expression of inflammatory genes [22,23]. In periodontally damaged tissues, a CoQ10 deficit was determined [21,24,25,26]. In contrast, oral application can increase CoQ10 concentration in the gingiva [27]. Besides topical administration, CoQ10 can be taken systemically as a dietary supplement [21].

A recent meta-analysis on CoQ10 as an adjunct to non-surgical periodontitis therapy gave the impression of a beneficial effect of its local administration on periodontal, gingival, and plaque parameters [18]. Nevertheless, no details about the performed treatments were provided. In addition, systemic CoQ10 administration was not included. However, that review admits that various aspects limit the meaning of its results, such as the quality of the studies, differences in application forms, and control groups [18]. In addition, no systematic review on different administration forms and formulations of CoQ10 has been available until now. Consequently, at the current timepoint, there are no evidence-based recommendations for the use of CoQ10 as an adjunct in non-surgical periodontitis therapy. Further clinical studies are necessary. For planning adequate study protocols, the completed studies should be considered. 

The present systematic review aims to collate the details of studies on CoQ10 as an adjunct to non-surgical periodontitis therapy. By this, it aims to provide recommendations for possible clinical protocols and especially for future study protocols on CoQ10 in initial periodontitis therapy. Therefore, RCTs on CoQ10 as an adjunct to SRP were identified.

## 2. Materials and Methods

The present systematic review was performed according to the Cochrane Handbook of Systematic Reviews of Interventions [28] and Preferred Reporting Items for Systematic Reviews and Meta-Analyses (PRISMA) guidelines [29]. The study protocol was developed in advance and was registered in the International Prospective Register of Systematic Reviews (PROSPERO, registration number CRD42021156887) [30].

### 2.1. Focused Question and Selection Criteria

The research question was the following: “What is the clinical effect of different study protocols of CoQ10 application as an adjunct to SRP?”. This question considered the following details according to PICOS: (P) Participants: patients of all age and gender with chronic periodontitis according to the criteria of Armitage 1999 [31] or periodontitis of stage II–IV according to Papapanou and Tonetti 2018 [32], with the actual need for periodontal treatment (PPD > 3 mm); (I) intervention: CoQ10 as an adjunct to SRP (close temporal connection); (C) comparison: SRP alone or SRP with placebo; (O) outcome: clinical periodontal parameters (primary outcome: periodontal probing depth, PPD [33]; secondary outcomes: bleeding on probing, BOP [34], and clinical attachment loss, CAL [33]; gingival indices, plaque indices); and (S) study design: randomized controlled clinical trials (RCTs). The language was restricted to English, German, French, Spanish, and Portuguese.

### 2.2. Search Strategy

A sensitive search strategy was followed. The following databases were searched up to 8 January 2022: MEDLINE via PubMed, MEDLINE via Web of Science, Web of Science Core Collection via Web of Science, Embase via Ovid, and Google Scholar. Unpublished data were searched on Cochrane Central Register of Controlled Trials (CENTRAL), WHO International Clinical Trials Registry Platform (ICTRP), and ClinicalTrials.gov. The grey literature was considered via searches in EASY (https://easy.dans.knaw.nl), GreyLit (https://greylit.org), and BASE (https://base-search.net). Boolean operators, truncations, and MESH terms were used if applicable. The search string for PubMed was “(“gingiv*”[All Fields] OR “periodont*”[All Fields] OR “scaling and root planning”[All Fields] OR “scaling and root planing”[All Fields] OR “root scaling” OR “dental scaling” OR “subgingival scaling” OR “probing depth”[All Fields] OR “pocket depth*”[All Fields] OR “attachment level”[All Fields] OR “attachment loss”[All Fields] OR “clinical attachment”[All Fields] OR “bleeding on probing”[All Fields] OR “Periodontal Diseases”[Mesh] OR “Periodontics”[Mesh] OR (“Journal of periodontology”[Journal]) OR (“Journal of periodontal research”[Journal]) OR (“Journal of clinical periodontology”[Journal])) AND (“Q10”[All Fields] OR “coq10”[All Fields] OR “CoQ”[All Fields] OR “coenzyme Q10”[All Fields] OR “ubiquinone”[All Fields] OR “ubiquinones”[All Fields] OR “ubiquinone 10”[All Fields] OR “ubiquinone”[MeSH Terms] OR “coenzyme Q10”[Supplementary Concept] OR “ubiquinone/therapeutic use”[MeSH Terms] OR “ubiquinol-10”[Supplementary Concept])”. Further search strings were modified according to provided search conditions of each database. Moreover, references of all included full texts as well as related review articles were screened for additional studies.

### 2.3. Study Selection

Two reviewers (C.L. and C.L.M.) independently screened titles and abstracts for eligibility according to the selection criteria. In case of an eligible title and abstract as well as if information relevant for the decision were missing, full texts were independently screened by the reviewers (C.L. and C.L.M.) with the registration of reasons in case of exclusion. Any disagreements were resolved by discussion with a third reviewer (G.S.).

### 2.4. Data Extraction

Two reviewers extracted data from the included studies. The following aspects were studied: characteristics of the studies including name of the authors, year of publication, country of investigation, observation period (in total and further timepoints of examinations), and study design (split mouth, SM, or full mouth, FM); characteristics of participants (number of participants in total, age and sex of the participants, and number of included teeth or sites), the included periodontal disease and periodontal inclusion criteria, systemic conditions of the participants (smoking status and systemic diseases), the study groups (treatment of study, control and additional groups, and number of participants or sites per group), and outcome measures with interest of the review; treatment modalities including product and dose and amount of the CoQ10 supplement, use of a placebo, details on the CoQ10 administration (application method, timepoints, duration of application, and instructions after application), and further procedures (pre-treatment, SRP protocol, and oral hygiene instructions); outcomes of the studies including baseline values of PPD and CAL, differences between baseline and study end for PPD and BOP and CAL, and the intergroup significances at the study’s end for all outcomes in regards to the interest of the review. Any disagreements were resolved by discussion until consensus was reached. In case of missing or unclear information, attempts were made to contact the authors. For this purpose, the corresponding authors of five studies [35,36,37,38,39] were contacted twice (November 2021 and January 2022) via the provided email addresses. All answers until November 2022 were considered. Two authors provided further information [35,36].

### 2.5. Risk of Bias Assessment

Two reviewers (C.L. and C.L.M.) independently assessed the risk of bias (RoB) of each included study using the Cochrane risk of bias assessment tool [40]. Potential bias was rated (high, low, or unclear) for the different domains (random sequence generation, allocation concealment, blinding of participants and personnel, blinding of outcome assessment, incomplete data outcome, selective reporting, and others). RoB domain-selective reporting was rated as low in case of accordance with the study protocol (if available) and if all applied main periodontal parameters (PPD, BOP, and CAL) were presented at the pre-specified timepoints. As potential other sources of bias, group differences in aspects with potential influence on periodontal parameters such as diabetes mellitus, smoking, use of mouthwashes, and baseline PPD were considered. Overall RoB was rated as low if all criteria were met, as unclear if at least one was not clearly reported, and high if one or more were not met. Any disagreements were resolved by discussion, consulting a third reviewer (D.Z.) if necessary. The results of the RoB assessment were presented graphically using Review Manager 5.4.1 from Cochrane [41].

## 3. Results

### 3.1. Study Selection (Figure 1)

From the initial 638 search results, title and abstract screening of 425 articles led to 31 articles undergoing full-text screening. Three additional studies were identified by references [39,42,43]. The reasons for exclusion in the full-text screening are presented in detail in the Supplementary Table S1. Finally, eighteen articles of seventeen studies were included.

**Figure 1:**
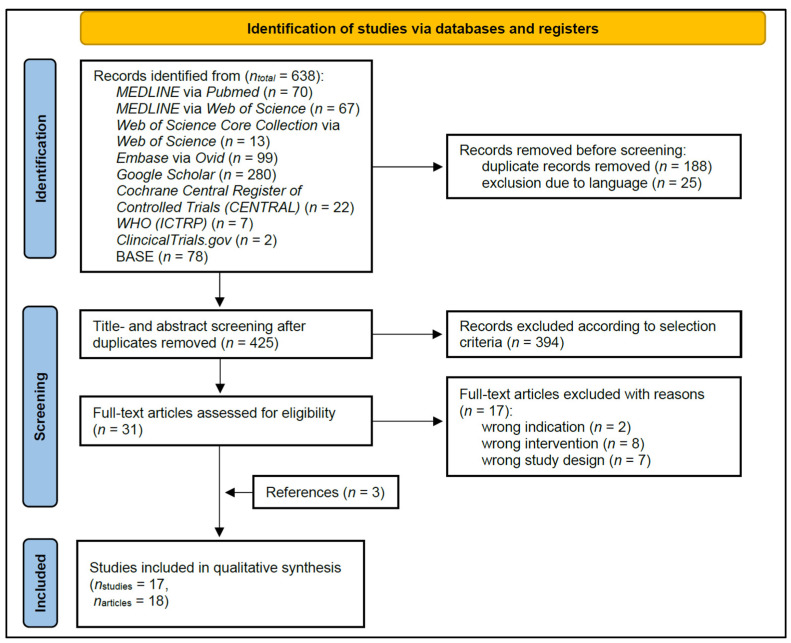
PRISMA flow diagram of the literature search, screening, and inclusion process, which included searches of databases and registers.

### 3.2. Characteristics of the Included Studies (Table 1)

The included articles were published from 2013 up to 2021 and were designed as monocentric studies mainly from India. The total observation period ranged from one to six months. Eight studies tested CoQ10 in an FM [35,36,37,39,42,44,45,46], eight in an SM study design [38,47,48,49,50,51,52,53], and one did not clearly report the design [43]. Only three studies applied a placebo [45,48,49], and all other studies were controlled against SRP alone. Some studies compromised a further study arm such as CoQ10 without SRP [35,38,51] or the application of other substances (SM: lycopene [46], tea-tree oil [49], and hyaluronic acid [53]; FM: doxycycline [36]). All studies examined PPD, and sixteen examined CAL [35,36,37,38,39,42,43,44,45,46,47,48,49,51,52,53] (in four studies measured as relative attachment loss, RAL [35,43,46,51]). Despite two studies using the expression “bleeding on probing” [51,52], no study focused on BOP as a percentage of bleeding sites on periodontal probing. The most popular gingival index was the Loe and Silness [54] index, and the most popular plaque index was the Silness and Loe [55] index (Table 1). Various articles neither precisely described the used indices nor presented a clear reference [36,37,47,49,50,51].

**Table 1:** Characteristics of the included studies.

Type of CoQ10 Administration Local/ Systemic)	Study	Country of Investigation	Observation Period Total (Time-Points)	Design SM/ FM	Participants *n* Total; Age Range (Mean ± SD); Sex f/m; *n_t/s_:* Teeth/sites	Periodontal Disease Periodontal Inclusion Criteria	Systemic Conditions Smoking Status; Systemic Diseases	Study Groups (*n_p_*: Participants, *n_s_*: Sites)	Outcome Measures with Interest of the Review
Local	Attia et al., 2016 [42]	Egypt	30 D	FM	*n* = 28; 29–46 (38 ± 6); 18 f, 10 m	CP, mild to moderate; PPD ≤ 6 mm, CAL ≤ 4 mm	Non-smokers; free from any systemic diseases (modified Cornell medical index)	Test group (*n_p_* = 14): SRP + CoQ10 Control group (*n_p_* = 14): SRP	PPD, CAL; LS; SL
	Barakat et al., 2019 [47]	Saudi Arabia	30 D	SM	*n* = 20; 25–60 (40 ± 4.33); 0 f, 20 m	CP, moderate	Systemically healthy (Cornell medical index)	Test group (*n_p_* = 20): SRP + CoQ10 Control group (*n_p_* = 20): SRP	PPD, CAL; BI; PI
	Chaudhry et al., 2014 [43]	India	30 D (15 D)	n.r.	20–50; both sexes; *n_s_* = 24	CP, generalized; PPD = 4–8 mm	No smokers; no systemic diseases, not pregnant, not lactating	Test group (*n_s_* = 12): SRP + CoQ10 Control group (*n_s_* = 12): SRP	PPD, RAL; LS; TGG
	Hans et al., 2012 [38]	India	6 W (3 W)	SM	*n* = 12; 22–55; both sexes; ≥4 sites per quadrant	CP, generalized; PPD = 4–8 mm; diagnosed clinically and radiographically	No smokers; no systemic diseases	Test group (*n_p_* = 12, *n_s_* ≥ 4): SRP + CoQ10 Control group (*n_p_* = 12, *n_s_* ≥ 4): SRP Additional group I (*n_p_* = 12, *n_s_* ≥ 4): CoQ10 topical Additional group II (*n_p_* = 12, *n_s_* ≥ 4): CoQ10 intrasulcular	PPD, CAL; LS, GBI; SL
	Jha et al., 2018 [46]	India	3 M (4 W)	FM/SM	*n* = 30; 22–55; both sexes	CP; PPD = 4–8 mm	No smokers; systemically healthy	Test group (*n_p_* = 15): SRP + CoQ10 Control group (*n_p_* = 15): SRP Additional group (*n_p_* = 15, SM with CoQ10): Lycopene	PPD, RAL; mSBIa
	Pranam et al., 2020 [48]	India	3 M (1 M)	SM	*n* = 16; 30–50 (42); 7 f, 9 m; *n_s_* = 64 (4 per participant)	CP, mild to moderate; PPD = 5–7 mm (in four non-adjacent interproximal sites)	No history of tobacco usage; no relevant medical history, not pregnant, not lactating	Test group (*n_s_* = 32): SRP + CoQ10 Control group (*n_s_* = 32): SRP + placebo	PPD, CAL; LS; TGG
	Raut et al., 2019 [44]	India	3 M (4 W)	FM	*n* = 40; 20–60 (37.4 ± 9.76)	CP, moderate to severe; PPD ≥ 5 mm, CAL ≥ 4 mm; diagnosed clinically and radiographically	Only current smokers with 10 cigarettes per day for min. 5 years; systemically healthy	Test group (*n_p_* = 20): SRP + CoQ10 Control group (*n_p_* = 20): SRP	PPD, CAL; mSBI; SL
	Raut et al., 2016 [49]	India	1 M	SM	*n* = 15; 20–60 (37.4 ± 9.76); 9 f, 6 m; ≥3 sites per participant	CP, moderate to severe, untreated; PPD > 5 mm, CAL > 4 mm; diagnosed clinically and radiographically	No smoking > 10 cigarettes per day; no systemic disease that affects the periodontium	Test group (*n_p_* = 15, *n_s_* = 15): SRP + CoQ10 Control group (*n_p_* = 15, *n_s_* = 15): SRP + placebo (methyl cellulose gel) Additional group (*n_p_* = 15, *n_s_* = 15): SRP + Tea-tree oil	PPD, CAL; SBI; SL
	Sale et al., 2014 [50]	India	4 W (2 W)	SM	*n* = 18; 20–55 (33.8); both sexes; min. 6 teeth per quadrant	CP; PPD ≥ 5 mm (in different quadrants); diagnosed clinically and radiographically	No smokers; no systemic diseases	Test group I (*n_p_* = 18): SRP + intrasulcular CoQ10 Test group II (*n_p_* = 18): SRP + topical CoQ10 Control group (*n_p_* = 18): SRP	PPD; LS, UBI; SL
	Salih et al., 2016 [51]	Iraq	6 W (3 W)	SM	*n* = 15; 33–55; min. 20 teeth	CP; PPD = 5–8 mm at min. 4 sites per quadrant CAL ≥ 1–2 mm	No smokers; systemically healthy	Test group (*n_p_* = 15, *n_s_* = 106): SRP + CoQ10 Control group (*n_p_* = 15, *n_s_* = 106): SRP Additional group (*n_p_* = 15, *n_s_* = 111): CoQ10	PPD, RAL; LS, UP; SL
	Shaheen et al., 2020 [52]	Egypt	7 W	SM	*n* = 15; 33–55; min. 20 teeth	Moderate periodontitis; PPD = 3–5 mm, CAL = 3–4 mm; diagnosed clinically and radiographically	No smokers; no systemic diseases	Test group (*n_p_* = 15): SRP + CoQ10 Control group (*n_p_* = 15): SRP	PPD, CAL; LS, PBI; SL
	Sharma et al., 2016 [53]	India	6 W (1 W, 2 W)	SM	*n* = 24; 25–55	CP, generalized; PPD ≥ 5 mm, BOP+	No systemic diseases	Test group (*n_p_* = 24, *n_s_* = 40): SRP + CoQ10 Control group (*n_p_* = 24, *n_s_* = 40): SRP Additional group (*n_p_* = 24, *n_s_* = 40): SRP + Hyaluronic acid	PPD, CAL; EIBI, GCCI; SL
Systemic	Darweesh et al., 2015 [36]	Egypt	3 M (1 M)	FM	*n* = 40; 37–55 (46.9 ± 5.7); 14 f, 26 m	CP, generalized moderate to severe; CAL > 3 mm	No smoking; no acute or chronic systemic disorders such as diabetes, hemorrhagic disorders cardio-vascular diseases, and conditions possibly affecting wound healing or interfering with the treatment or affecting patient’s compliance	Test group (*n_p_* = 10): SRP + CoQ10 Control group (*n_p_* = 10): SRP Additional group I (*n_p_* = 10): SRP + Doxycycline Additional group II (*n_p_* = 10): SRP + CoQ10 + Doxycycline	PPD, CAL; GI, BI; PI
	Mani et al., 2013 [39]	India	4 M (2 M)	FM	*n* = 100; 18–55	CP, generalized	No smokers, no tobacco in any form; no diabetes, arthritis, heart diseases, obesity, neurological disorders, or diseases with possible effects on the immune system	Test group I (*n_p_* = 25): SRP + CoQ10 (with chlorine dioxide toothpaste and mouthwash), Test group II (*n_p_* = 25): SRP + CoQ10 (with conventional toothpaste and mouthwash) Control group I (*n_p_* = 25): SRP (with chlorine dioxide toothpaste and mouthwash Control group II (*n_p_* = 25): SRP (with conventional toothpaste and mouthwash)	PPD, CAL; LS; TGG
	Pandav et al., 2021 [35]	India	3 M (6 W)	FM	*n* = 60; 30–60	CP; PPD = 3–5 mm, BOP+	No smokers; systemically healthy	Test group (*n_p_* = 20): SRP + CoQ10 Control group (*n_p_* = 20): SRP Additional group (*n_p_* = 20): CoQ10	PPD, RAL; LS
	Saini et al., 2014 [37]	India	4 M (2 M)	FM	*n* = 50; 18–55; 21 f, 29 m	CP, generalized	No smokers, no tobacco in any form; No diabetes, arthritis, heart diseases, obesity, neurological disorders, or diseases with possible effects on the immune system	Test group (*n_p_* = 25): SRP + CoQ10 Control group (*n_p_* = 25): SRP	PPD, CAL; GIa; PI
	Shoukheba et al., 2019 [45]	Egypt	6 M (4 W, 3 M)	FM	*n* = 30; 30–50; 18 f, 12 m	CP, generalized moderate; CAL = 4–5 mm	No smokers, no tobacco in any form; diabetes mellitus type II (HbA1c: 6–8%) and no further systemic diseases	Test group (*n_p_* = 15): SRP + CoQ10 Control group (*n_p_* = 15): SRP + Placebo	PPD, CAL; LS, MS

BI, “bleeding index” without detailed description or clear reference; CAL, clinical attachment loss [33]; CP, chronic periodontitis; D, days; EIBI, Eastman interdental bleeding index [56]; f, female participant; FM, full-mouth study design; GBI, gingival bleeding index according to Ainamo and Bay 1975 [34]; GCCI, gingival color-change index [57]; GI, “gingival index” without detailed description or clear reference; Gia, measured on 16, 12, 24, 32, 36, 44; LS, gingival index according to Loe and Silness 1963 [54]; m, male participants; M, months; MS, sulcus bleeding index according to Mühlemann and Son 1971 [58]; mSBI, modified sulcus bleeding index according to Mombelli 1987 [59]; mSBIa, index measured on first + second premolar, first + second molar; n.r., not reported; PBI, “bleeding on probing” with described scoring system equivalent to papillary bleeding index according to Saxer and Mühlemann 1975 [60]; PI, “plaque index” without detailed description or clear reference; PPD, periodontal probing depth [33]; RAL, relative attachment level (partially describing the use of occlusal stents); SBI, “sulcus bleeding index” without detailed description or clear reference; SL, plaque index according to Silness and Loe 1964 [55]; SM, split-mouth study design; SD, standard deviation; SRP, scaling and root planning; TGG, plaque index according to Turesky–Gilmore–Glickman modification of Quigley–Hein index 1970 [61]; UBI, “gingival bleeding index” and “modified sulcular bleeding index” without detailed description or clear reference; UP, “bleeding on probing” without detailed description or clear reference, suspected not to be the percentage of bleeding sites on probing; W, weeks.

#### 3.2.1. Study Samples (Table 1)

In total, over 500 subjects were included in all studies, with a maximal group size of 25. The age of all participants ranged from 18 years to 60 years, with only seven studies reporting a mean age [36,42,44,47,48,49,50]. In ten studies, both genders were included [36,37,38,42,43,45,46,48,49,50]; one study examined only male subjects [47], and six studies did not specify [35,39,44,51,52,53].

Periodontal inclusion criteria differed widely, reaching from mild to severe CAL and partly only minimum or only maximum values for PPD. Likewise, the inclusion criteria of participants’ systemic conditions varied (eleven studies included only patients without any systemic conditions [35,38,42,43,44,46,47,50,51,52,53], one without systemic diseases affecting the periodontium [49], one “without relevant medical history” [48], and one without conditions possibly affecting wound healing or interfering with the treatment or affecting patient’s compliance [36]; three studies excluded specific predefined systemic conditions [36,37,39]; one focused on patients with diabetes [45]). One study explicitly examined smokers [44], one included up to 10 cigarettes per day [49], nine excluded smokers [35,36,38,42,43,46,50,51,52], four excluded any tobacco use [37,39,45,48], and two did not specify [47,53]. All besides two studies mentioned the use of antibiotics as an exclusion criterion [35,37,38,39,42,43,44,45,46,47,48,49,50,51,53]. Use of mouth rinses was excluded in four [35,42,45,48] and standardized in two studies [37,39]. Eleven studies excluded previous periodontal therapy during the last six months [37,39,42,43,44,45,46,47,48,50,53] and two studies in the previous two [49]/three [52] months, and four did not specify [35,36,38,51].

#### 3.2.2. Treatment Modalities (Table 2)

Twelve studies with local (twelve times intrasulcular [38,42,43,44,46,47,48,49,50,51,52,53] and one time topical [50]) and five studies with systemic [35,36,37,39,45] CoQ10 administration were included. Ten studies used the product Perio Q gel (PerioQ INC, Manchester, USA) [38,42,43,44,47,48,49,50,51,53]. There was a lack of information regarding the individually applied amount of gel or dose of CoQ10. Local CoQ10 administration was performed once [38,43,44,46,47,48,49,51] or several times in a period up to 15 days [42,50,52,53]. Four studies applied periodontal dressing [44,47,49,53], while six studies restricted food and/or oral hygiene after application [42,46,48,50,51,52]. Systemic CoQ10 was applied twice daily [35,36,45] or three times daily [37,39] (total daily dose if reported: 60 mg [35,36,45]) for six weeks [35] or three [36,45] or four months [37,39] in accordance to observation timepoints. SRP was performed in up to three visits.

**Table 2:** Treatment modalities including details on the provided coenzyme Q10 (CoQ10) supplement, placebo as a control, CoQ10 administration, and scaling and root planing (SRP) procedure. Missing information in the table indicates that no according information was reported in the manuscripts.

Type of CoQ10 Administration (Local/Systemic)	Study (Name, Year)	CoQ10 Supplement Product; Dose; Amount as Reported	Placebo-Controlled (Yes, No)	CoQ10 Administration (Application Method; Timepoints, Duration of Application; Instructions after Application)	Procedures (Pre-Treatment; SRP Protocol; Oral Hygiene Instructions)
Local	Attia et al., 2016 [42]	Perio Q gel, PerioQ INC, Manchester, USA; gel; 1:9	No	Intrasulcular, isolation (cotton rolls), drying (paper points) + blunted needle tip; at day 0–day 7–day 15; restriction for eating, spitting, drinking for one hour, brushing, and flossing for four hours after application	SRP (in 3 visits); instructions for plaque control regimen and oral hygiene provided at each appointment, no use of mouthwashes (exclusion criteria)
	Barakat et al., 2019 [47]	Perio Q^TM^ gel, Hamilton, USA; gel; 0.2 ml	No	Intrasulcular, one time, directly after SRP, periodontal dressing for 1 week	SRP (in 2 visits) using ultrasonic scaler and Gracey curettes
	Chaudhry et al., 2014 [43]	Perio Q^TM^ gel, Hamilton, USA; 0.2 ml	No	Intrasulcular, syringe; one time, directly after SRP	SRP using ultrasonic scaler
	Hans et al., 2012 [38]	PerioQ gel, PerioQ INC, Manchester, USA; gel; 1:9	No	Intrasulcular, drying (paper points), syringe with irrigation needle (Max-i-probe, Dentsply, USA); one time, one day after SRP	SRP (one day after recording of clinical parameter) with ultrasonic scaler and hand instruments
	Jha et a. 2018 [46]	Gel; 2%; 0.1 ml	No	Intrasulcular; syringe + blunt cannula; one time, directly after SRP; refrain from chewing hard or sticky foods, brushing near the treated areas or using any interdental aids for 1 week	SRP performed until the root surface was considered smooth and clean by the operator
	Pranam et al., 2020 [48]	Perio Q_10_, Perio Inc, United States	Yes	Intrasulcular, drying (paper points), 2 mL syringe with intrasulcular applicator tip, withdrawn extruding till the superior portion of the pocket, one time, directly after SRP; avoidance of dental floss or interdental aids or mouth rinses	SRP at baseline; oral hygiene instructions: modified bass brushing technique, no dental floss or interdental aids, no use of mouth rinses (exclusion criteria)
	Raut et al., 2019 [44]	Perio Q gel, PerioQ Inc., Manchester, USA; gel; 1:9	No	Intrasulcular, special needles, periodontal pack for 7 days; one time, directly after SRP	SRP at baseline
	Raut et al., 2016 [49]	Perio Q^®^ gel, PerioQ Inc., Manchester, USA; gel; 1:9	Yes (methyl-cellulose gel)	Intrasulcular, special needles, periodontal pack for 7 days; one time, directly after SRP;	SRP at baseline
	Sale et al., 2014 [50]	Perio Q^TM^ gel; gel; 1:9	No	Intrasulcular: special needles; topical: tip of the applicator completely soaked in gel; every alternate day for one week; restriction for eating, spitting, and drinking for 1 h after application	SRP at baseline
	Salih et al., 2016 [51]	Perio Q gel; gel; 1:9; total 1 mL, each pocket 0.1–0.3 ml	No	Intrasulcular, isolation (cotton rolls), drying (air, paper points), syringe + blunted needle, removing gel excessing from pocket; one time one hour after SRP if bleeding the next day; instruction to avoid spitting, washing, eating, and drinking for 2 h after application, pause of toothbrush and interdental aids the day after the gel application	Supragingival scaling; motivation and instruction in initial visit
	Shaheen et al., 2020 [52]	NMQ10 (self-produced; thermo-reversible carrier system); gel; 5%; 0.1 mL per study side	No	Intrasulcular; syringe + atraumatic needle; each alternate day for one week; restriction for rinsing, drinking for 1 h after application	Full-mouth SRP; oral hygiene instructions
	Sharma et al., 2016 [53]	Perio Q10 gel, PerioQ Inc., Manchester, USA; gel; 1:9	No	Intrasulcular, wide-gauge needle, gel slightly overflowing, periodontal pack; three times: after SRP, 1 week–2 weeks;	-
Systemic	Darweesh et al., 2015 [36]	CoQ10, BioMérieux, France; capsules; 30 mg	No	Systemic; twice a day, 3 months	SRP using hand and ultrasonic instruments (Hu-Friedy EMS Piezon^®^, Chicago, IL, US), hand instruments
	Mani et al., 2013 [39]	Oxyfresh CoQ10 Complex; dietary supplement	No	Systemic; three times a day, 4 months	SRP using EMS ultrasonic scaler; tooth brushing: twice daily 5 min with modified bass method technique (technique demonstrated to each subject) with the provided similar medium-bristle toothbrushes, toothpaste: Oxyfresh Power Paste (with chlorine dioxide)/Pepsodent (conventional), mouth rinse: twice daily (5 mL in quantity for 1 min), Oxyfresh Power Rinse (with chlorine dioxide)/Listerine (conventional)
	Pandav et al., 2021 [35]	Recharje Forte, Troikaa Pharmaceuticals Ltd. Uttarakhand, India; capsules; 30 mg	No	Systemic; twice a day, 6 weeks	No use of mouth rinse was permitted
	Saini et al., 2014 [37]	Nutritional supplement of CoQ10, Oxyfresh^®^ Company; nutritional supplement	No	Systemic; three times a day, 4 months	SRP at baseline, using Electro Medical Systems ultrasonic scaler; tooth brushing: twice daily for 5 min with modified bass method technique (technique demonstrated to each subject) with the provided similar medium-bristle toothbrushes, conventional toothpaste, conventional mouth wash
	Shoukheba et al., 2019 [45]	CoQ10, MEPACO-MEDIFOOD, Enshas El Raml-Sharke-ia-Eygpt; capsules; 30 mg	Yes (oral placebo capsule)	Systemic; twice a day, 3 months	Full-mouth SRP in two sessions at an interval of 1 week, polishing, no SRP at recall visits; twice daily brushing technique with interproximal plaque control, no mouthwash

#### 3.2.3. Outcomes (Table 3)

PPD reduction ranged from 0.20 mm to 2.95 mm. Significant group differences for PPD favoring the test group were reported in five of twelve studies on local administration [43,44,47,51,52] and in two of five studies on systemic administration [35,45]. One study reported a significant difference favoring the control group [37]. No study reported adverse effects.

**Table 3:** Outcomes of the included studies with the difference between baseline and study end (∆) for periodontal probing depths (PPD), bleeding on probing (BOP), and clinical attachment loss (CAL) if applicable. Intergroup significances at the study end are presented for all outcomes (additionally for gingival index and plaque index). Bold marks significant differences.

Type of CoQ10 Administration (Local/ Systemic)	Study	Groups	Primary Outcomes			Secondary Outcomes					
			PPD			BOP	CAL			Gingival Index	Plaque Index
			Baseline (mm)	∆ PPD (mm) *	Intergroup Significance*		Baseline (mm)	∆ CAL (mm)*	Intergroup Significance *	Intergroup Significance *	Intergroup Significance *
Local	Attia et al., 2016 [42]	Test	4.22 ± 0.36	0.90	NSSD *p* = 0.103	N/A	2.35 ± 0.96	0.65	NSSD *p* = 0.312	NSSD *p* = 0.601	NSSD *p* = 0.635
		Control	4.27 ± 0.44	0.72			2.33 ± 0.47	0.50			
	Barakat et al., 2019 [47]	Test	3.76 ± 0.49	0.59	**SSD f.t.** ***p* < 0.001**	N/A	2.76 ± 0.47	0.46	**SSD f.t.** ***p* = 0.006**	**SSD f.t.** ***p* = 0**	**SSD f.t.** ***p* = 0**
		Control	3.83 ± 0.55	0.33			2.83 ± 0.49	0.32			
	Chaudhry et al., 2014 [43]	Test	5.50 ± 5.52	2.00	**SSD f.t.** ***p* = 0.018**	N/A	9.50 ± 1.087	2.08	**SSD f.t.** ***p* = 0.031**	**SSD f.t.** ***p* = 0.000**	NSSD *p* = 0.557
		Control	5.58 ± 0.52	1.33			9.42 ± 0.900	1.54			
	Hans et al., 2012 [38]	Test	4.97 ± 0.23	1.14	NSSD *p* = 0.90	N/A	2.25 ± 0.48	0.33	NSSD *p* = 0.78	LS: NSSD *p* = 0.62 **GBI: SSD f.t.** ***p* < 0.05**	NSSD *p* = 0.22
		Control	4.75 ± 0.34	1.02			2.08 ± 0.32	0.38			
	Jha et al., 2018 [46]	Test	5.39 ± 0.31	2.26	n.r.	N/A	6.55 ± 0.26	2.42	n.r.	n.r.	N/A
		Control	6.39 ± 0.48	2.20			6.21 ± 0.43	1.81			
	Pranam et al., 2020 [48]	Test	5.47 ± 0.56	1.16	NSSD *p* = 0.321	N/A	6.13 ± 0.61	1.25	NSSD *p* = 0.448	NSSD *p* = 0.210	NSSD *p* = 0.110
		Control	5.13 ± 0.42	1.09			6.16 ± 0.62	1.19			
	Raut et al., 2019 [44]	Test	6.42 ± 0.60	2.83	**SSD f.t.** ***p* < 0.001**	N/A	5.59 ± 0.63	2.52	**SSD f.t.** ***p* < 0.001**	**SSD f.t.** ***p* = 0.0004**	NSSD *p* = 0.329
		Control	6.33 ± 0.66	1.95			5.46 ± 0.60	1.75			
	Raut et al., 2016 [49]	Test	5.69 ± 0.83	2.95	n.r.	N/A	5.94 ± 0.80	2.33	n.r.	NSSD	NSSD
		Control	5.10 ± 0.68	0.50			5.22 ± 0.64	0.45			
	Sale et al., 2014 [50]	Test I	6.33 ± 1.09	2.61	NSSD *p* = 0.965	N/A	N/A	N/A	N/A	LS: NSSD *p* = 0.518, **UBI: SSD f.c.** ***p* = 0.031**	**SSD f.t.** ***p* < 0.0001**
		Test II	5.72 ± 0.57	2.00							
		Control	5.00 ± 0.84 ^c^	1.34							
	Salih et al., 2016 [51]	Test	6.20 ± 0.62	1.01	**SSD f.t.**	N/A^a^	7.20 ± 0.62	1.45	**SSD f.t.**	n.r.	n.r.
		Control	6.40 ± 0.66	0.65			7.50 ± 0.66	0.75			
	Shaheen et al., 2020 [52]	Test	2.66 ± 0.42	1.09	**SSD f.t.** ***p* < 0.05**	N/A^b^	2.47 ± 0.36	1.04	**SSD f.t.** ***p* < 0.005**	**LS: SSD f.t.** ***p* < 0.005,** **PBI: SSD f.t.** ***p* < 0.005**	**SSD f.t.** ***p* < 0.05**
		Control	2.51 ± 0.42	0.66			2.38 ± 2.04	0.55			
	Sharma et al., 2016 [53]	Test	5.53 ± 0.59	2.33	NSSD	N/A	4.53 ± 1.26	1.66	NSSD	EIBI: NSSD, GCCI: NSSD	NSSD
		Control	5.60 ± 0.87	2.23			4.50 ± 1.01	1.75			
Systemic	Darweesh et al., 2015 [36]	Test	4.57 ± 0.44	1.17	NSSD	N/A	4.79 ± 0.52	1.40	NSSD	**GI: SSD f.t.** ***p* = 0.0001**, **BI: SSD f.t. *p* = 0.0003**	**SSD f.t.** ***p* = 0.0001**
		Control	4.78 ± 0.41	1.17			4.90 ± 0.50	1.25			
	Mani et al., 2013 [39]	Test I	n.r.	n.r.	n.r.	N/A	4.08 ± 1.19	1.20	n.r.	n.r.	n.r.
		Control I	n.r.	n.r.			4.37 ± 1.17	0.41			
		Test II	n.r.	n.r.	n.r.		5.64 ± 0.72	1.12	n.r.	n.r.	n.r.
		Control II	n.r.	n.r.			5.64 ± 0.60	0.24			
	Pandav et al., 2021 [35]	Test	3.6 ± 0.68	1.35	**SSD f.t.** ***p* = 0.018**	N/A	6.70 ± 0.96	1.22	NSSD *p* = 0.153	NSSD *p* = 0.489	N/A
		Control	3.5 ± 0.36	1.05			6.72 ± 0.92	0.87			
	Saini et al., 2014 [37]	Test	5.28 ± 1.16	0.20	**SSD f.c.** ***p* < 0.01**	N/A	5.64 ± 0.72	1.12	**SSD f.t.** ***p* < 0.01**	**SSD f.c.** ***p* < 0.01**	**SSD f.c.** ***p* < 0.01**
		Control	6.74 ± 0.79	0.66			5.64 ± 0.60	0.24			
	Shoukheba et al., 2019 [45]	Test	5.66 ± 0.72	1.20	**SSD f.t.** ***p* = 0.008**	N/A	4.33 ± 0.48	0.40	**SSD f.t.** ***p* = 0.002**	**LS: SSD f.t.** ***p* = 0,** **MS: SSD f.t.** ***p* = 0**	N/A
		Control	5.46 ± 0.74	0.40			4.53 ± 0.51	−0.13			

∆, difference of mean between baseline and study end; * refers to the last follow-up of the study; BI, “bleeding index” without detailed description or clear reference; BOP, bleeding on probing according to Ainamo and Bay 1975 [34]; CAL, clinical attachment loss [33]; CoQ10, coenzyme Q10; EIBI, Eastman interdental bleeding index [56]; GBI, gingival bleeding index according to Ainamo and Bay 1975 [34]; GCCI, gingival color-change index [57]; GI, “gingival index” without detailed description or clear reference; LS, gingival index according to Loe and Silness 1963; MS, sulcus bleeding index according to Mühlemann and Son 1971 [58]; N/A, not applicable; n.r., not reported; NSSD, no statistically significant difference; PBI, “bleeding on probing” with described scoring system equivalent to papillary bleeding index according to Saxer and Mühlemann 1975 [60]; PPD, periodontal probing depth [33]; SSD f.t., statistically significant difference favoring test group; SSD f.c., statistically significant difference favoring control group; UBI, “gingival bleeding index” and, respectively, “modified sulcular bleeding index” without detailed description or clear reference. ^a^ The “bleeding on probing” mentioned in the study is suspected not to be the percentage of bleeding sites on probing according to Ainamo and Bay 1975; ^b^ the “bleeding on probing” mentioned in the study is described as a scoring system equivalent to papillary bleeding index according to Saxer and Mühlemann 1975, and results are presented at gingival index as PBI; ^c^ significant group difference (*p* < 0.0001) at baseline with lower PPD values in control group.

### 3.3. Risk of Bias

**Figure 2:**
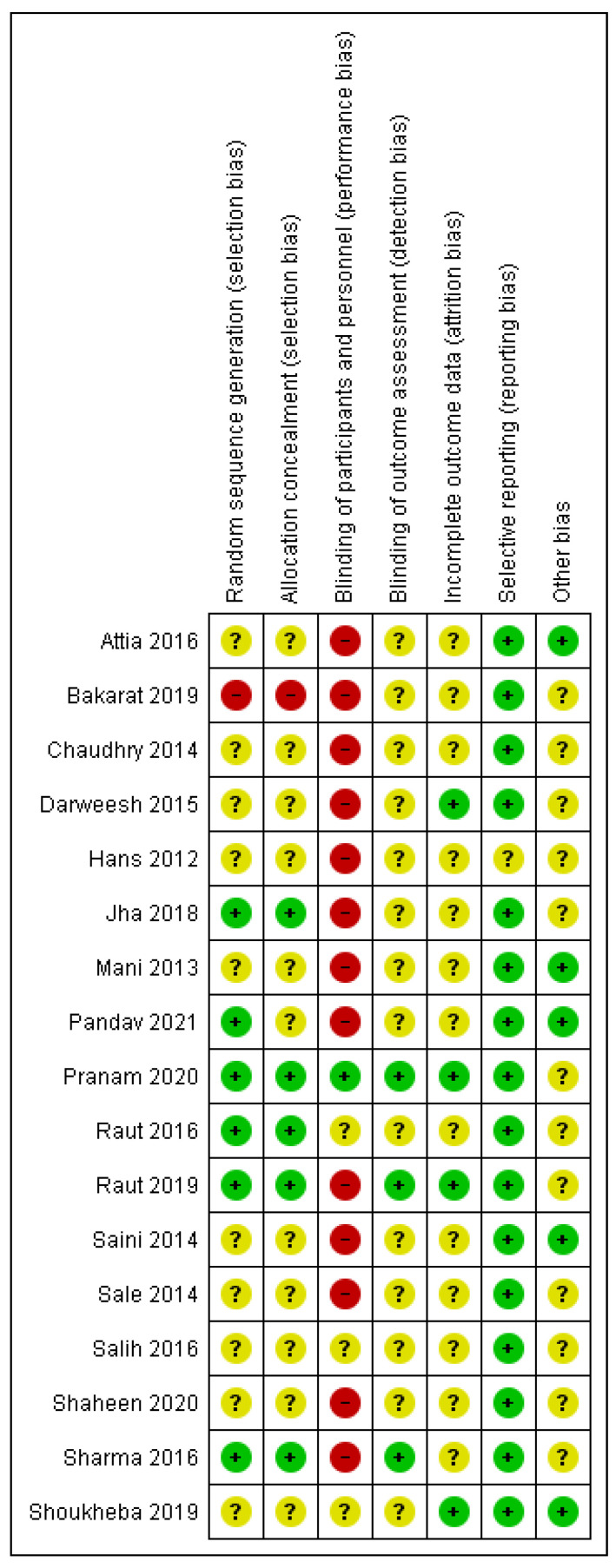
Summary of risk of bias analysis: review authors’ judgment about the different domains for each included study. A green circle (+) indicates a low risk of bias, a yellow circle (?) an unclear risk of bias, and a red circle (−) a high risk of bias in the respective domain. As potential other sources of bias, the following were considered: potential group differences regarding diabetes, smoking habits, and mouthwashes.

The RoB rating for each study is presented in Figure 2. In general, many domains were rated with an unclear RoB due to insufficient method description. Overall, thirteen studies were classified with high [35,36,37,38,39,42,43,44,46,47,50,52,53] and four with unclear RoB [45,48,49,51].

Only six articles reported the random sequence generation [35,44,46,48,49,53] and only five allocation concealment [44,46,48,49,53]. One study predetermined the right side as the test side and the left one as the control side [47]. Only one study fulfilled blinding of participants and personnel [48], and only three studies reported blinding of outcome assessment [44,48,53]. In addition, the completeness of outcome data was reported by only four studies [36,44,45,48]. No selective reporting was observed, but one study was rated unclear (possibly imprecise method description) [38]. For five studies, no other sources of bias were identified [35,37,39,42,45], while for twelve studies, at least one other source of bias remained unclear [36,38,43,44,46,47,48,49,50,51,52,53].

## 4. Discussion

This systematic review identified 17 applicable RCTs examining the adjunctive use of CoQ10 to SRP in non-surgical periodontitis therapy. In general, the available protocols were very heterogeneous. First of all, this applies for the CoQ10 administration. Twelve studies used local and five systemic administrations. The individually applied CoQ10 amounts are mainly unclear. Some studies applied 60 mg per day as a nutritional supplement [35,36,45]. Such an amount could be recommendable, as it had been shown that 50 mg CoQ10 per day as capsules increases the activity of succinate dehydrogenase-coenzyme Q10 reductase in the gingiva [27]. In addition, a positive effect on markers of inflammation and MMPs [62] and oxidative stress [63] in blood has been demonstrated in the context of other diseases. However, here, the doses were even higher, with at least 90 mg per day. The four studies reporting amounts for locally applied CoQ10 used many times lower doses of CoQ10 at one time (per pocket: 0.1–0.3 mL 1:9 [43,47,51] and 0.2 mL 2% [46]; total: 1 mL 1:9 [51]). In addition, especially for local administration, the applied CoQ10 administration periods were short (Table 2). Thus, the possibility of an effect on periodontal healing is questionable. Regarding the question of the recommendable administration mode (local versus systemic), no studies with direct comparison were available. For both strategies, some studies revealed significant differences, while others did not (Table 3). One study provided a direct comparison of intrasulcular and topical application on the gingiva but without significant difference (Table 3) [50]. Application strategies of CoQ10 should be developed carefully including the investigation of the effect on both local and systemic CoQ10 levels and inflammatory parameters. It is important for all studies on CoQ10 to report in detail the formulation, dose, and application strategy of the CoQ10 product. In addition, more details of the CoQ10 administration, such as draining, management of bleeding, and application of periodontal dressing, must be taken into account. At a later timepoint, if the efficacy of different strategies were verified, direct comparisons in clinical studies should be carried out to determine the ideal protocol. Four studies applied periodontal dressing [44,47,49,53], and six studies restricted food and/or oral hygiene after application [42,46,48,50,51,52]. Furthermore, the protocols of the periodontal therapy (SRP) itself are reported insufficiently and differed widely, too (Table 2).

Regarding the question of the outcome measurement, the anti-inflammatory properties of CoQ10 [22,23] could lead to a greater reduction of the periodontal inflammation in the test groups. Consequently, indicators of the absence of inflammation are of interest. For this aim, BOP and PPD as indicators for periodontal stability [64] should be considered. No studies with BOP as outcome were available (Table 3). PPD is the other major criterion for periodontal stability [64] and must therefore be considered as the most meaningful parameter in the available studies. Most studies with local administration had a maximum observation period of two and those with systemic administration of maximum four months.

Currently, another systematic review on adjunct CoQ10 gel in initial periodontitis therapy has been published with data assessment up to 2020 [18]. This review indicates significant group differences for PPD, CAL, “bleeding index”, “gingival index”, and “plaque index”. For PPD, it estimated a WMD of about 1 mm, and it concludes that the use of CoQ10 in combination with SRP could improve periodontitis. However, critical appraisal is necessary. The results of these meta-analyses must be interpreted with caution because there are several limitations: One point is the included studies (one study with “gingivitis or slight periodontitis” and studies investigating CNBC gel, which is a combination preparation). In addition, the rating of the RoB in that systematic review must be critically discussed (placebo gel as a parameter for a high risk at the category “other bias” and rating as unclear RoB at “blinding of participants and personnel” despite no placebo and no special strategies for blinding). The meta-analyses are based on studies of poor quality. Moreover, the strategies to deal with the generally huge heterogeneity of the pooled studies questions the informative value of the meta-analyses (observation periods between 1 and 48 weeks, equating full-mouth studies to four sites, and different indices). Especially, the homogenization of different indices for the “plaque index” and the “bleeding index” to one scale must be criticized, as the methods of these indices are very different, and a simple conversion is not possible. This applies even more as some studies do not even present the used method clearly. Furthermore, the definition of “bleeding index” versus “gingival index” in the above review remains unclear and is misleading. It is important to emphasize that none of the studies investigated the percentual periodontal parameter bleeding on probing. Moreover, and not least, the missing information of the applied CoQ10 amounts or doses leads to the pooling of data of unknown heterogeneity. In the opinion of the authors, the high RoB, the low quality, and the great heterogeneity of the studies deny any quantitative synthesis. A rating of the quality of evidence is missing for these meta-analyses and would probably be very low according to the GRADE [65] approach. Consequently, despite the calculated results, the conclusion would rather be that the evidence is very uncertain about an additional benefit of the local use of CoQ10 as an adjunct to SRP. These limitations question the positive appraisal of the efficacy of CoQ10 in the above review despite the use of RCTs and supposedly high class of evidence. The revealed limitations of the included studies should be considered for tailoring future protocols.

Regarding other adjuncts to SRP, meta-analyses identified significant MD for PPD between 0.2 and 0.4 mm for local antimicrobials [12], about 0.5 mm for systemic antibiotics [10], up to 0.5 mm for probiotics [16,17], and about 0.5 mm for the antioxidant omega-3 [66]. Generally, several studies on CoQ10 revealed similar MD (Table 3). However, the study results were contradictory, and only about half of the studies verified a significant difference between test and control groups. In general, the results of the studies must be discussed critically regarding RoB and study quality (see below). All in all, it is unclear if CoQ10 might have similar potential as an adjunct for non-surgical periodontitis therapy, and further studies should investigate this topic. However, SRP alone is already efficient for reducing PPD. Consequently, the clinical relevance of such adjuncts is questionable, especially as the determined benefits are small or partly because no benefit was even found at all. Nevertheless, for example, systemic antibiotics are at least recommended for special patient groups [67]. Because of their disadvantages, the search for alternatives is important. Interestingly, in periodontitis-risk patients, an even greater efficacy can be suspected, as the adjunctive effects for PPD were high both in smokers [44] (MD = 0.9 mm, *p* < 0.001) and diabetics [45] (MD = 0.8 mm, *p* = 0.008) (Table 3) despite longer observation periods of three or six months, respectively [44,45]. Thus, special patient groups with immunological imbalance, such as smokers [68,69] and diabetics [70], might be an interesting target group and should be considered explicitly in future studies.

To the best knowledge of the authors, this is the first systematic review presenting in detail the different protocols of both systemically and locally applied CoQ10 in non-surgical periodontitis therapy. The methods of such a systematic review guarantee high transparency and avoid bias. They enable a quick comprehensive overview of the available information. The study protocol was developed and registered in advance. A sensitive search strategy according to the PICOS criteria considered both local and systemic administration, and a comprehensive literature search could be accomplished. Notably, the consideration of Google Scholar, the search for unpublished data and grey literature, as well as cross-checking of references allow the consideration of this review as a comprehensive collation of the currently available evidence for this research question. The study designs were inspected in detail, and all outcomes were considered with transparent separation of the different indices for plaque and gingivitis. In case of missing information, attempts were made to contact the authors. Nevertheless, some limitations must be addressed: Regarding the study selection, several search results had to be excluded in the title and abstract screening due to language restrictions. Despite the sensitive search strategy, only a few studies were available, and the total number of included patients remained low. No study investigated the inflammatory parameter BOP (Table 3). The reporting quality was low, and most of the missing information could be completed neither by study protocols nor by request to the authors. Consequently, unclear reporting with missing or inconsistent information even after requests to the authors led to exclusion of several studies (Supplementary Table S1). Nevertheless, even in the included studies, important factors as the exact amounts of CoQ10 are missing. Consequently, various issues remained unspecified, and RoB was evident. Nearly all studies had a high risk for performance bias and placebo effects because no blinding of the participants and personnel had been performed (Figure 2). In view of the presence of these potential sources of bias, some exceptionally great MD must be critically discussed. In addition, the included studies show great heterogeneity. Notably, the CoQ10 administration itself differed widely (e.g., timepoint of application, number of applications, and use of periodontal pack; Table 2) as well as SRP. In particular, a large variety of factors about the included age, sex, and periodontitis diagnosis must be stated. Different PPD were investigated in the different studies, but none considered different ranges separately. All in all, the available studies are not comparable. Furthermore, study quality is low: Small sample sizes of the included studies, short observation periods (Table 1), no description of calibration, and no registered study protocols must be mentioned.

The practical implications of this systematic review must be discussed. The available evidence does not allow recommendations for the use of CoQ10 in non-surgical periodontitis therapy. Further high-quality RCTs with precise reporting according to Consort Guidelines are necessary. Adequate study protocols should be planned carefully considering the lessons of the presented studies. Especially for ethical reasons, futile studies must be avoided. The following implications for future study protocols became apparent: Inclusion and exclusion criteria should be defined exactly to attain a homogenous cohort. In particular, all diseases and conditions directly or indirectly affecting the periodontium should be excluded (e.g., diabetes mellitus, diseases or medication affecting the immune system or manual skills, and smoking). Age, sex, and number of teeth should be considered for restrictions or matching and should be reported per group. Furthermore, the periodontal disease itself and included PPD should be defined. Group sizes should be based on a calculation using the available results of previous studies. Outcomes should especially include BOP and PPD (indicators for periodontal stability) [64] and gingival indices to investigate the supposed anti-inflammatory effect. Side effects should be registered. The exact definition of the CoQ10 administration, including exact product, dose (mg), application methods, duration, and timepoints of administration are of primary importance. Furthermore, the SRP as the periodontal treatment should be described precisely. Full-mouth strategies simplify the study protocol due to a clear starting point. Both participants and dentists (treating and investigating) should be blinded (double-blind studies) to avoid performance and detection bias. To enable such blinding, a placebo is necessary that would also avoid the detection of placebo effects. Pretreatments, instructions after treatment, and oral hygiene instructions should be defined. Especially, antiseptics and antibiotics should be excluded. Furthermore, observation periods should be extended to allow estimation of the long-term efficacy and, consequently, the clinical relevance.

## 5. Conclusions

The current evidence is very uncertain regarding an additional benefit of the adjunct use of CoQ10 in non-surgical periodontitis therapy. The available studies are heterogeneous in their methods, showing contradictory results and a low methodological quality. Until now, no statements regarding the effectiveness on periodontal stability (PPD and BOP) and improvement of the clinical situation are possible. Further long-term and high-quality RCTs are necessary and should consider the recommendations of this review.

## Data Availability

Not applicable.

## Supplementary Materials

The following supporting information can be downloaded at: https://www.mdpi.com/article/10.3390/nu15071585/s1, Table S1: Reasons for exclusions of studies in full-text screening. References [27,71,72,73,74,75,76,77,78,79,80,81,82,83,84,85,86] are cited in the Supplementary Materials.
