# Supplementary material for: Systematic Review on Protocols of Coenzyme Q10 Supplementation in Non-Surgical Periodontitis Therapy
Source: Nutrients. 2023 Mar 24;15(7):1585. doi: 10.3390/nu15071585 (PMC10096526; doi:10.3390/nu15071585)
Supplement: Supplementary file 1 [file nutrients-15-01585-s001.zip › nutrients-2221426-supplementary.pdf]

Systematic Review on Protocols of Coenzyme Q10 Supplementation in Non-Surgical Periodontitis Therapy

Cordula Leonie Merle, Carina Lenzen, Gerhard Schmalz and Dirk Ziebolz

Supplementary Table S1. Reasons for exclusions of studies in full text screening.

Study	Reason for Exclusion in the Full Text Screening
Anwaristi et al., 2020	wrong study design: no control group
Bhalla et al., 2019	wrong study design: no control group without active substances
Chug et al., 2020	wrong intervention: CoQ10 administration one month after SRP in patients who did not respond
Folkers et al., 1977	wrong study design: narrative review
Hanioka et al., 1994	wrong intervention: start of CoQ10 administration before SRP
Lister et al., 1995	wrong study design: letter to the editor
Manthena et al., 2015	wrong indication: not clearly periodontitis patients
Mathews-Brzozowska et al., 2007	wrong study design: no control group
Matsumura et al., 1973	wrong intervention: no SRP
Nakamura et al., 1973	no intervention
Pitale et al., 2012	wrong indication: generalized gingivitis or slight periodontitis (1-2 mm CAL)
Rasperini et al., 2019	wrong intervention: combination preparation (micronutrient complex tablets); start of CoQ10 administration before SRP
Roopa et al. 2014	wrong intervention: combination preparations (CNBC gel)
Saini et al., 2011	wrong study design: narrative review
Wilkinson et al., 1975	wrong study design: no control group,<br>wrong intervention: CoQ10 administration after SRP in patients who were assigned for surgical correction
Wilkinson et al., 1976	wrong intervention: no SRP
Zaki et al., 2012	wrong intervention: no SRP

References

Anwaristi, A.Y. Effect of coenzyme-q10 in the post-curettage against probing depth, relative attachment loss, and bleeding on probing. J. Syiah Kuala Dent. Soc. 2022, 5, 66–69, https://doi.org/10.24815/jds.v5i2.20015.

Bhalla, A.; Jithendra, K.D.; Shailendra, S. Co-Enzyme Q10:-Another Armour For Novel Periodontal Therapy. 2019.

Chug, A.; Shukla, S. Placement of Sticky Bone™ in patients with generalized periodontitis previously treated with coen-zyme Q10. J. Contemp. Dent. Pract. 2020, 21, 156–160.

Folkers, K.; Watanabe, T. Bioenergetics in clinical medicine-X. Survey of the adjunctive use of coenzyme Q with oral thera-py in treating periodontal disease. J. Med. 1977, 8, 333–348.

Hanioka, T.; Tanaka, M.; Ojima, M.; Shizukuishi, S.; Folkers, K. Effect of topical application of Coenzyme Q10 on adult per-iodontitis. Mol. Asp. Med. 1994, 15, s241–s248, https://doi.org/10.1016/0098-2997(94)90034-5.

Lister, R.E. Coenzyme Q10 and periodontal disease. Br. Dent. J. 1995, 179, 200–201.

Manthena, S.; Rao, M.V.R.; Penubolu, L.P.; Putcha, M.; Harsha, A.V.N.S. Effectiveness of CoQ10 Oral Supplements as an Ad-junct to Scaling and Root Planing in Improving Periodontal Health. J. Clin. Diagn. Res. JCDR 2015, 9, ZC26-8.

Matthews-Brzozowska, T.; Kurhañska-Flisykowska, A.; Wyganowska-Swiatkowska, M.; Stopa, J. Healing of periodontal tissue assisted by coenzyme Q10 with vitamin E—Clinical and laboratory evaluation. Pharm. Rep. 2007, 59 (Suppl. S1), 257–260.

Matsumura, T.; Saji, S.; Nakamura, R.; Folkers, K. Evidence for enhanced treatment of periodontal disease by therapy with coenzyme Q. Int. J. Vitam. Nutr. Res. 1973, 43, 537–548.

Nakamura, R.; Littaru, G.P.; Folkers, K; Wilkinson, E.G. Deficiency of Coenzyme Q in Gingiva of Patients with Periodontal Disease. J. Int. Vitaminol. Nutr. 1973, 43, 84–92.

Pitale, U.; Khetarpal, S.; Peter, K.; Pal, V.; Verma, E.; Gupta, P. Evaluation of efficacy of coenzyme Q 10 in management of gingivitis & slight periodontitis - a clinical study. Int. J. Curr. Pharm. Res. 2012, 4, 33–38.

Rasperini, G.; Pellegrini, G.; Sugai, J.; Mauro, C.; Fiocchi, S.; Mora, P.C.; Dellavia, C. Effects of food supplements on periodontal status and local and systemic inflammation after nonoperative periodontal treatment. J. Oral Sci. 2019, 61, 213–220, https://doi.org/10.2334/josnusd.18-0048.

Roopa, D.A.; Gupta, R.; Gupta, I.; Chauhan, S.; Pandey, A.; Sharma, N.K. Clinical evaluation of topical application of CNBC gel (Coenzyme Q10) in chronic periodontitis patients. J. Dent. Res. Updates 2014, 1, 13–17.

Saini, R. Coenzyme Q10: The essential nutrient. J. Pharm. Bioallied Sci. 2011, 3, 466–467.

Wilkinson, E.G.; Arnold, R.M.; Folkers, K.; Hansen, I.; Kishi, H. Bioenergetics in clinical medicine. II. Adjunctive treatment with coenzyme Q in periodontal therapy. Res. Commun. Chem. Pathol. Pharmacol. 1975, 12, 111–123.

Wilkinson, E.G.; Arnold, R.M.; Folkers, K. Bioenergetics in clinical medicine. VI. Adjunctive treatment of periodontal disease with coenzyme Q10. Res. Commun. Chem. Pathol. Pharmacol. 1976, 14, 715–719.

Zaki, N.M. Site-specific delivery of the Nutraceutical COQ10 for periodontal therapy. Int. J. Pharm. Pharm. Sci. 2012, 4, 717–723.
